# Physiotherapy in Bangladesh: Inequality Begets Inequality

**DOI:** 10.3389/fpubh.2018.00080

**Published:** 2018-03-23

**Authors:** Firoz Ahmed Mamin, Rieke Hayes

**Affiliations:** Department of Physiotherapy, Bangladesh Health Professions Institute, Centre for Rehabilitation of the Paralysed, Savar, Dhaka, Bangladesh

**Keywords:** Bangladesh, sustainable development goals, universal health coverage, rehabilitation, physiotherapy

## Abstract

**Background:**

The demand for health services in developing countries often outweighs provision. This article describes the present condition of physiotherapy in Bangladesh. Physiotherapy is not recognized as a profession by the government. There is no single registration and regulation body. The health-related and economic benefits of physiotherapy are not felt by the majority of Bangladeshi citizens.

**Areas covered:**

The burden of disease is changing, and Bangladesh needs a profession that specializes in physical rehabilitation to face these challenges. This article outlines the benefits to patients and the wider economy from a broad physiotherapy regime for all Bangladeshi citizens. It describes the many barriers the profession faces.

**Conclusion:**

Physiotherapy is efficacious in many post-trauma situations and long-term conditions. Economic evidence supports the provision physiotherapy as a cost-effective treatment which should be considered as part of the provision of a universal health-care service. Official recognition of the protected “physiotherapist” title and a single registration and regulation agency are recommended.

## Introduction

Physiotherapists are specialists in developing and maintaining people’s ability to move and function throughout all stages of their lives. They promote healthy lifestyles, treat, and prevent many problems caused by illness, injury, pain, disease, age, and inactivity. They work with people affected by a range of conditions including arthritis, back pain, lung disease, spinal cord injuries, falls, stroke, incontinence, fractures, burns, and mental health issues. The ultimate goal of rehabilitation is to provide the individual with the best possible opportunity for full and effective participation and inclusion in society. Physiotherapists work in many diverse settings, including hospitals, emergency medical teams, community settings, hospices, nursing homes, health centers, education, and research.

In many developing countries, physiotherapy is poorly understood and therefore lacks the recognition, funding, and resources to be utilized effectively. Bangladesh is no exception to this.

Bangladesh became an independent state in 1971. The National Institute of Traumatology and Orthopedic Rehabilitation (NITOR) was established to treat and rehabilitate those wounded during the war of liberation with Pakistan. This demand for rehabilitation specialists led to the development of the first physiotherapy course in the country in 1973. This course only saw two classes graduate before it was discontinued (1).

Through involvement with Dhaka University, the Bangladesh Health Professions Institute (BHPI) and NITOR developed a BSc degree in physiotherapy in the 1990s. This course was a 4-year program with over 1,000 clinical placement hours, followed by a 1-year compulsory internship, a model which is still in use today.

To date, there are seven institutions which provide a BSc Physiotherapy qualification: BHPI, NITOR, Dhaka Institute of Health and Technology, Rajshahi Institute of Health and Technology, State College Health Sciences, SAIC Institute of Medical Technology, and Gono Bishwabidyalay. In addition to this, both the BHPI and Gono Bishwabidyalay provide an MSc Physiotherapy program.

There is a severe shortage of physiotherapists to serve the huge population of Bangladesh. Whereas approximately 54.7 thousand physiotherapists were registered in the United Kingdom in 2017 (2) (a population of around 65 million), only an estimated 1.7 thousand physiotherapists exist in Bangladesh today (a population of around 160 million). This estimate is based on the number of graduates reported since the 1990s. Ironically, the employment opportunities for these physiotherapists are very limited. Many graduates set up private practices and few find work in private hospitals, nongovernmental organizations (NGOs), or seek employment abroad. As the profession has no formal register, no exact figures currently exist on how many of these graduates are currently practicing as physiotherapists.

This bleak situation can be largely attributed to one major issue: BSc physiotherapists are not formally recognized by the government. As a result, they are not employed to work clinically in the public health sector. Despite BSc qualifications being issued by government institutions, only diploma physiotherapists (known as health or medical technologists) are employed to work in public hospitals at this time (3). This work is carried out under the instruction of a physiatrist (a doctor trained in physical medicine), rather than a physiotherapist, and is largely restricted to electrotherapy in a musculoskeletal outpatient setting.

Physiotherapy lacks an official definition as a discipline among other health services in both the Bangladesh National Health Policy 2011 and the Health, Population and Nutrition Sector Development Program 2011–2016. Acute physical rehabilitation has almost been absent under the Ministry of Health and Family Welfare while long-term rehabilitation has been provided under the Ministry of Social Welfare and NGOs (3). There is a lack of coordination between these health and social welfare agencies (4).

Bowing to the growing pressure from student groups, which culminated in a hunger strike in 2010, the government allocated land and funding to create the Bangladesh College of Physiotherapy in Dhaka in 2011 (5, 6). The building never began construction, however, and the land plot is currently being used as an urban slum (7). The National Health Policy 2011 made a single reference to physiotherapy, stating that the profession required further development. It contained no plan as to how it should be developed nor was follow-up action taken after the publishing of this policy. On the other hand, the policy called for further investment in alternative therapies such as unani, ayurvedic, and homeopathy training and recruitment. These professions are formally recognized by the government, registered, trained in state colleges, and are employed in public hospitals (8). Tackling the perception that physiotherapy and physical rehabilitation are not essential to health care is a pivotal step to addressing this poor investment from the government.

## Title and Registration Issues

By denying the profession recognition at a government level, the state has also rendered it free from governance. The title of physiotherapist is neither protected nor regulated. This is understandably a concern, as a regulatory body is in place to protect the public as much as they protect the profession itself.

Professional bodies do exist, but their role is limited to advocacy, peer support, and professional development. At present, two such groups exist: the Bangladesh Physiotherapy Association, which is a member of the World Confederation of Physical Therapists, and also the Bangladesh Physical Therapy Association (1, 9). Membership with either, though encouraged, is not obligatory. They represent, but they do not regulate the profession in Bangladesh.

The lack of registration also means that little information exists about the availability, accessibility, and quality of the physiotherapy service. Through a register of accredited professionals that meet a set of standards in education, skill, conduct, and behavior, it would be an offense, punishable by law, to use a protected title inappropriately; thus potentially dangerous practice could be prevented. A regulatory body aims to uphold high standards of education and training in order to ensure good practice within the profession. Such safeguards exist to ensure the professionalism and accountability of the physiotherapy profession, thus protecting the public from malpractice.

Professional registration and regulation is a crucial step to legitimizing and empowering the physiotherapy profession in Bangladesh.

## The Bangladeshi Health Care

Though exact figures for the population vary, the consensus is that the population currently sits at around 160 million people, making it one of the most densely populated countries in the world at 1,252 people per km^2^ (10).

An estimated 70% of the population live in rural areas. However, Bangladesh is going through rapid urbanization generating cities of unprecedented density and congestion (11). Dhaka, its capital, is considered the most densely populated city in the world with 44,500 people per km^2^ (12).

Though considered a developing, lower middle-income country, progress has been made toward eradicating poverty over the past 30 years, reducing poverty from 57% in 1991–1992 to 25% in 2015 (13). Vision 2021 sets the poverty target at 14% which, if achieved, would result in Bangladesh becoming a middle-income country by 2021 (14).

Bangladesh has the lowest spending on health per capita in South Asia. In the 2015–2016 fiscal year, only 4.3% of the national budget was allocated to the health sector equating to 32 United States Dollar (USD) per capita. This is considerably below the World Health Organization (WHO) recommended level of 15%, which is a per capita spending of 54 USD (15). Despite this, Bangladesh has achieved remarkable accomplishments in many areas of health in the face of its economic poverty.

Maternal mortality decreased by 75% since 1980 (16). Infant mortality more than halved since the 1990s (11). Life expectancy now averages 72 years, and it is estimated that almost 19% of the population will be over 60 years of age by mid-century (17).

Many of these improvements have been attributed to the use of pluralism (18). Following the formation of state, the government encouraged an environment in health care, and social welfare could be supplemented considerably by outside donors, NGOs, and private enterprises.

The government provides hospitals at district and subdistrict (Upazila) levels in Bangladesh. Although the public health system is, in principle, free to the population, it does incur costs indirectly through medication, laboratory tests, surgical instruments, and transport costs. Staffing for public health services is limited. There are 4.9 doctors and 2.9 nurses registered for every 10,000 residents in Bangladesh; however, only 1.43 and 1.05, respectively, per 10,000 population work in public health care (19). The health worker density needed to address the sustainable development goals (SDGs) is estimated at 4.45 doctors, nurses, and midwives per 1,000 population (20). The issues within the public health sector are considerable: critical staff are absent, facilities are inadequate, quality of staffing is poor, patients often travel great distances to facilities, there are informal fees, poor or absent communication, and long waiting times. Poor governance and accountability within the health sector exacerbate these problems (21). These issues and the perception of poor and unreliable services may explain why those who can afford it often seek private health-care services (22).

In addition to severe shortages, Bangladesh’s health workforce is also characterized by its inequitable and uneven geographical distribution. The already-limited medical and nursing staff, whether private or public, are concentrated predominantly in urban areas. The vacancy rate for all medical staff is low in Dhaka but often rises significantly with an increasing distance from the capital. On average, the vacancy rate was 43.3% for physicians in 2011 with the highest vacancy reported in Gaibandha district at 80.5% (21). This leaves over 70% of Bangladesh’s population, who live in rural areas, with very minimal access to free public health care. These people are also among the poorest in the country. Frontline health services in rural settings thus tend to be informal “village doctors” and unregistered drug vendors. The proliferation of unregulated, often low-quality, and high cost private practitioners is fast becoming a concern for public safety (23).

The private sector dominates health care. In 2011, 80% of hospitals were private for profit, accounting for 50% of all hospital beds in Bangladesh, and this figure continues to grow every year (24). Around 50% of doctors and 42% of nurses in Bangladesh work exclusively for the private sector, with many additional staff working in both private and government hospitals (25). This duality has been known to affect public sector services where informal payments by patients for free or unnecessary services add up to 80% of what is spent more formally on fees in private sector facilities (18). Almost two-thirds of health-care costs in a household expenditure are in the private sectors (18). Even in urban areas, however, where the availability of health care is significantly greater, hospitals and services are often financially and socially unattainable to the population (26). Accessing the nearest clinic, which is usually private, can be achieved a few times in life because the majority of Bangladeshis simply cannot afford to. However, these are the people who need it the most, for poor health and disability can cause poverty and poverty can exacerbate disability. For a poor population with growing long-term conditions, there is a need for accessible and affordable public health care (11).

It is estimated that 4,000 NGOs, both national and international organizations, provide health-care services in areas which remain underfunded and understaffed by the state (27). These areas include, but are not limited to, women’s health, rehabilitation, orthotics and prosthetics, education, and care for people with disabilities.

## Provision of Rehabilitation Services

Rehabilitation services in public and private health care do not exist in Bangladesh, and this paucity is being only addressed, by some degree, by NGOs. As a result, physiotherapy is not sufficiently included in health policies by the government and is both under-resourced and underfunded. The government recruits no qualified physiotherapists in the public health sector. Stroke, fractures, amputees, and spinal cord injuries are just a few of the many conditions that receive neither inpatient nor community rehabilitation through the public sector. Many of these patients are simply discharged home once medically fit without any follow-up or rehabilitation which could reduce their dependence and help integrate them into society. This is a significant oversight by a government that is committed to implementing the global 2030 SDGs. Universal health coverage is a prominent part of the SDGs and aspires to ensure that all people can use the promotive, preventative, curative, rehabilitative, and palliative health services they need, while also ensuring that the use of these services does not expose the user to financial hardship (28). The staffing crisis compounds the public health service inefficiencies. In order to reach its goal by 2030, the country will have to improve the availability and the skill mix of its health workforce (29), and acknowledging the specialty of physiotherapist in providing rehabilitation is an essential step.

The burden of disease in Bangladesh is shifting. The population is growing in size, and there is also a shift in not just an aging but an old population (17). Increased urbanization, matched by a rapidly developing infrastructure, is increasing the number of vehicles on the road and is shifting work sector toward the garments industry. The prevailing health and disease profiles are changing in Bangladesh, and the health sector needs to change with it.

According to the WHO, 15% of the world’s population suffers some form of disability and 80% of these can be found in developing countries (30, 31). Gupta et al. estimates that 92% of the disease burden in the world is related to causes requiring health professionals associated with physical rehabilitation (32).

Noncommunicable diseases (NCDs), including injuries, now account for 60% of deaths in Bangladesh (33). A 2010 government survey found a high incidence of smoking (26%), diabetes (3.9%), hypertension (17.9% stage I, 5.5% stage II), and low physical activity (27%) within Bangladesh (34). These are all contributing factors to NCDs such as cancer, chronic lung diseases, stroke, and other cardiovascular disease, which fall under the top five causes of death in the country (33). The size and skill of the current health workforce is inadequate to deal with an increasing prevalence of NCDs which require a huge preventative health care. Rehabilitation specialists such as physiotherapists remain a largely untapped resource in Bangladesh. Participation in physical exercise can help prevent and reduce many chronic diseases, and physiotherapists can advise on the management and prevention of future health problems associated with NCDs. According to the WHO estimate 2011, the cost of preventative action would be US$11.4 billion per year across all low- and middle-income countries which equates to the annual cost of less than US$1 per person living in a low-income country (35). Addressing NCDs should be the top priorities not only for health but for rapid economic development of the country through saving scarce resources.

Manual labor and the garments industry are noted for their poor work conditions. In a small-scale study, almost 62% of workers in a garments factory suffered some form of musculoskeletal problems (36). According to the Global Burden of Disease 2015 study, the health problems, which resulted in the two highest causes for disability in Bangladesh, were back and neck pain and “other musculoskeletal problems” (37). Physiotherapy is effective in reducing both acute and chronic pain, limiting the risk of further disability and contributes to improved physical function, including return to work and recreational activity (38–40).

Rising motorization in South Asia has not been accompanied sufficiently by improvements in road safety strategies. According to a WHO report, 1.3 million deaths globally are due to road traffic accidents (RTAs), 90% of which occur in low- to middle-income countries (41). In addition to this, between 20 and 50 million people are estimated to have nonfatal injuries, with many of those who survive left with temporary or permanent disabilities. According to the Bangladesh Health and Injury Survey 2016, there are over 23,000 road traffic fatalities a year in Bangladesh, equating to 64 deaths a day. Over 3.4 million people a year suffer nonfatal injuries as a result of RTAs with over 80,000 experiencing permanent disability (42). One of the recommendations of the study is to strengthen rehabilitation interventions for these victims.

In addition to the physical and emotional toll on those affected, disabilities can also incur a considerable economic loss to victims, their families, and the nation as a whole. Losses arise from the cost of treatment, reduced or lost wages, and for family members who need to take time off work to care for the disabled. A study published in April 2017 found that residents in Bangladesh faced serious difficulties with health-care financing; 1 in 10 households incurred financial catastrophe and 1 in 20 non-poor households became poor due to health-care costs (43). Those who are poor are more likely to become disabled, and those who are disabled are more likely to become poor.

Besides the direct benefits physiotherapy can have to the health and quality of life of a patient population, rehabilitation can also have a positive impact on the economy. A period of structured rehabilitation for injuries and musculoskeletal problems can reduce the degree of impairment, restoring function, improving recovery time, and return to work, thus reducing the financial burden (44, 45). At present, the health system does not have the capacity to answer the needs of these patients, leaving them without a proper treatment, at risk of further complications and hamper their reintegration into society. Physiotherapy plays an integral role to promoting and improving health in a population.

With this in mind, it is vital that the government addresses the paucity in a workforce that is skilled in addressing rehabilitation needs, thus improving quality of life and enabling those with disabilities to be able to contribute to the economy. The sustainable development agenda cannot be effectively achieved without addressing the unmet needs for rehabilitation services (46).

## Conclusion

Bangladesh is moving toward universal health coverage. Health workforce planning must address how to meet the twin goals of increased accessibility and provision of high-quality care. Three elements must now be considered. First, the recognition of the protected title of physiotherapist along international standards, in tandem, a merged, single registration and regulation agency. Second, a commitment to the provision of physiotherapist posts in proportion to the expansion of a universal health-care mandate. Third, an evaluation in the Bangladeshi context of the explicit link between physiotherapy provision and the improved quality of life, integration into society, return to employment, and reduction of financial burden associated with illness.

## Author Contributions

All authors contributed equally in the research and write-up to this article.

## Conflict of Interest Statement

The authors declare that the research was conducted in the absence of any commercial or financial relationships that could be construed as a potential conflict of interest.

## Abbreviations

BHPI, Bangladesh Health Professions Institute; NCD, noncommunicable disease; NGO, nongovernmental organization; NITOR, National Institute of Traumatology and Orthopedic Rehabilitation; RTA, road traffic accident; SDG, sustainable development goals; USD, United States Dollar; WHO, World Health Organization.
